# Phosphate solubilizing bacteria stimulate wheat rhizosphere and endosphere biological nitrogen fixation by improving phosphorus content

**DOI:** 10.7717/peerj.9062

**Published:** 2020-05-04

**Authors:** Yongbin Li, Qin Li, Guohua Guan, Sanfeng Chen

**Affiliations:** State Key Laboratory of Agrobiotechnology and College of Biological Sciences, Beijing, China

**Keywords:** Diazotrophic bacteria, Phosphate solubilizing bacteria, *Paenibacillus*, Biological nitrogen fixation, Wheat

## Abstract

Phosphate (P) availability often limits biological nitrogen fixation (BNF) by diazotrophic bacteria. In soil, only 0.1% of the total P is available for plant uptake. P solubilizing bacteria can convert insoluble P to plant-available soluble P (ionic P and low molecular-weight organic P). However, limited information is available about the effects of synergistic application of diazotrophic bacteria and P solubilizing bacteria on the nitrogenase activity of rhizosphere and *nifH* expression of endosphere. In this study, we investigated the effects of co-inoculation with a diazotrophic bacterium (*Paenibacillus beijingensis* BJ-18) and a P-solubilizing bacterium (*Paenibacillus* sp. B1) on wheat growth, plant and soil total N, plant total P, soil available P, soil nitrogenase activity and the relative expression of *nifH* in plant tissues. Co-inoculation significantly increased plant biomass (length, fresh and dry weight) and plant N content (root: 27%, shoot: 30%) and P content (root: 63%, shoot: 30%). Co-inoculation also significantly increased soil total N (12%), available P (9%) and nitrogenase activity (69%) compared to *P. beijingensis* BJ-18 inoculation alone. Quantitative real-time PCR analysis showed co-inoculation doubled expression of *nifH* genes in shoots and roots. Soil nitrogenase activity and *nifH* expression within plant tissues correlated with P content of soil and plant tissues, which suggests solubilization of P by *Paenibacillus* sp. B1 increased N fixation in soils and the endosphere. In conclusion, P solubilizing bacteria generally improved soil available P and plant P uptake, and considerably stimulated BNF in the rhizosphere and endosphere of wheat seedlings.

## Introduction

Biological nitrogen (N) fixation (BNF) is the main N input in many ecosystems (Berthrong et al., 2014). In this process, diazotrophic bacteria convert atmospheric N_2_ into ammonia (Vitousek et al., 2002), and fix 50–70 Tg microbial N in agricultural systems annually (Herridge, Peoples & Boddey, 2008). In low phosphorus (P)-content soil, P controls the rates of heterotrophic BNF (Reed, Cleveland & Townsend, 2007, 2011) and often limits BNF in the rhizobial symbiosis (Kleinert et al., 2014).

Although there are large amounts of organic and inorganic P in soils, only 0.1% total P is available for plants (Zou, Binkley & Doxtader, 1992), resulting in huge demand for chemical P fertilizer. Frequent application of chemical P fertilizer is expensive, and damages ecosystems by causing problems such as the loss of soil fertility (Gyaneshwar et al., 2002) and eutrophication (Schindler et al., 2008). In practice, chemical P fertilizer utilization efficiency is less than 30% (Sharma et al., 2013), because of fixation of P by free metal ions in soil (Hagin & Hadas, 1962; Sharma et al., 2013). Leaching and run-off also lead to loss of P fertilizer (Sashidhar & Podile, 2009). P ore, a finite resource, is primary original material of chemical P fertilizer. This high quality P ore may run out within 50–100 years (Cordell, Drangert & White, 2009). Khan, Zaidi & Wani (2007) reported that mobilization of insoluble P accumulated in agricultural soils would meet the demand of P for agriculture for a century. Application of P solubilizing bacteria can mobilize P and provide growers an alternative in agriculture that minimizes the application of chemical P fertilizer (Sharma et al., 2013) and maximize BNF rates. Arbuscular mycorrhiza stimulate BNF of symbiotic diazotrophic bacteria by improving P acquisition in the legume–rhizobial symbiosis (Pueschel et al., 2017).

Free-living *Paenibacillus* strains have potential as biofertilizers due to their capacity to form endospores, which can survive for long time periods under adverse conditions (Grady et al., 2016). Previously, we isolated the diazotrophic *P. beijngensis* BJ-18 from the rhizosphere of wheat. This plant growth promoting rhizobacteria (PGPR) exhibits high nitrogenase activity (Wang et al., 2013) and multiple antagonistic activities against plant pathogens (Xie et al., 2016). *P. beijingensis* BJ-18 produces 3-indoleacetic acid and can increase wheat yield by 27% in the field (Shi et al., 2016), and promote the growth of tomato seedlings (Xie et al., 2016). Observations of GFP-labelled *P. beijingensis* BJ-18 with confocal laser scanning microscopy shows that this diazotroph effectively colonizes the endosphere of roots, stems, and leaves (Li et al., 2019a). We also previously isolated *Paenibacillus* sp. B1 from the rhizosphere of maize. This PGPR can solubilize organic P (lecithin) and inorganic P and promote the growth and improve nutrition of maize and tomato. Obervations of GFP-labbled *Paenibacillus* sp. B1 shows that this P-solubilizing strain effectively colonizes maize root surface and in epidermal and cortical tissues (Li et al., 2017).

To test the hypothesis that these two PGPR, with complementary traits, show synergy, a factorial wheat (*Triticum aestivum* L.) pot experiment with different inoculation treatments was conducted. We investigated the effects of experimental treatments on (1) host plant biomass, (2) rhizosphere soil total N, available P and nitrogenase activity, (3) plant tissue total N, total P and relative expression of *nifH* and (4) the Pearson’s correlation coefficient between the nitrogenase activity/*nifH* expression and P nutrition of the soil/plant tissues.

## Materials and Methods

### Bacteria strains and growth conditions

*P. beijingensis* BJ-18 (accession number: JN873136) was isolated from rhizosphere soil of wheat (Wang et al., 2013). *Paenibacillus* sp. B1 (accession number: KY111475), isolated from the maize rhizosphere, is a novel species with P solubilizing ability and produce IAA (Li et al., 2017). *P. beijingensis* BJ-18 and *Paenibacillus* sp. B1 were inoculated individually in Erlenmeyer flasks (250 mL) containing 100 mL of Luria Bertani broth and cultured at 30 °C overnight. After growth, the bacterial cells were harvested by centrifugation at 4,000×*g* for 5 min and diluted with deionized water to 10^8^ cells/mL.

### Plant culture and collection

Seedling growth assays were performed in plastic pots (diameter of 9 cm; height of 12 cm) filled with 1 kg low nutrition-content soil. The collection and physicochemical properties of soil were described by Li et al. (2019b). The treatments contained: (1) wheat seedling inoculated with *Paenibacillus* sp. B1 (B1) (2) wheat seedling inoculated with *P. beijingensis* BJ-18 (BJ-18) (3) wheat seedling inoculated with *Paenibacillus* sp. B1 and *P. beijingensis* BJ-18 (v:v = 1:1; Mix) (4) non-inoculated wheat seedlings as a negative control (CK) and (5) wheat seedlings inoculated with *P. beijingensis* BJ-18 and watered with nutrient solution containing soluble P (BJ-18+P) as a positive control. Plants were irrigated with N-free nutrient solution (0.65 mM MgSO_4_, 2 mM CaCl_2_, 0.75 mM K_2_SO_4_, 0.1 mM KCl, 0.25 mM KH_2_PO_4_, 0.2 mM Fe-EDTA, 1 × 10^−3^ mM MnSO_4_, 1 × 10^−3^ mM ZnSO_4_, 1 × 10^−4^ mM CuSO_4_, 6.2 × 10^−6^ mM Na_2_MoO_4_, 1 × 10^−3^ mM H_3_BO_3_) in the presence or absence of KH_2_PO_4_ where applicable (43 mL per pot).

Plump seeds of wheat “Jimai 22” (Shandong Runfeng Seed Industry Co., Ltd., Gaomi, Shandong, China) were soaked in 10% sodium hypochlorite for 10 min, and rinsed thrice with sterilized water. The sterilized wheat seeds were placed on the sterile plates containing moist filter papers and cultivated for 3–5 days at room temperature (25 °C). After germination, vigorous and homogeneous wheat seedlings were chosen. For inoculation, the wheat seedlings were soaked in bacteria suspension (10^8^ cells/mL) for 30 min to facilitate colonization. For CK treatment, the plant seedlings were soaked in sterilized deionized water. Then the inoculated and non-inoculated seedlings were respectively transplanted into pots (3 hills per pot and 15 seedlings per hill). Sixteen millilitres of the bacteria suspension were applied to pots containing inoculated seedlings and 16 mL of sterile water was applied to pots containing non-inoculated seedlings on day 7. All treatments had five replicates. Pots were placed in the greenhouse under optimum conditions (15 h light/9 h dark cycle, 25–30 °C day/15–20 °C night temperature and 40% day/60% night humidity). The seedlings were watered every 5 days.

On day 35 after transplanting, the samples of wheat were collected. For collection of rhizosphere soil, the wheat seedling was uprooted carefully, and the intact root was shaken gently to remove the loosely adhering soil. Then the tightly adhering soil was gathered as rhizosphere soil. Shoot and root samples were separated and the lengths of the roots and shoots measured immediately. Then other root and shoot samples were dried for dry weight analysis. Then, the samples were ground for plant total P and total N content. The remaining plant samples were kept in a freezer at −80 °C. Chlorophyll content of seedlings was determined at 8:30–9:30 AM on the harvest day using the SPAD-502 chlorophyl meter (Minolta Camera Co. Ltd., Tokyo, Japan).

### Elemental analyses of soil and plant tissue

The oven-dried samples were weighed and ground into fine powders. An appropriate amount of soil and plant samples was digested by an H_2_SO_4_–H_2_O_2_ mixture at 370 °C and then total N was determined using the modified Kjeldahl method (Baker & Thompson, 1992). Plant P content was determined using the standard vanadomolybdate method (Hanson, 1950). Available P of soil was extracted with resin and measured following (Hedley & Stewart, 1982).

### Biological N_2_ fixation assay

The acetylene reduction method was used to detect nitrogenase activity of rhizosphere soil (Jean et al., 2013; Perakis, Pett-Ridge & Catricala, 2017), with slight modification. Two grams of rhizosphere soil were added to a 26 mL glass jar fitted with septa and flooded with sterile deionized water to 240% moisture content. The jars were incubated at 28 °C in the dark for 48 h prior to replacing 10% (v/v) of the headspace with acetylene. After incubation for 2 h, 100 μL of headspace air were collected to measure ethylene production by gas chromatograph (GC-2010 Plus; Shimadzu, Kyoto, Japan). The nitrogenase activity was expressed as nmol ethylene g^−1^ soil h^−1^.

### Quantitative real-time PCR analysis of nifH in plant roots and shoots

Transcript levels of *nifH* gene, one of *nif* genes coding Fe protein of nitrogenase, were quantified by RT-qPCR. RNAiso Plus reagent was used to extract plant total RNA according to the manufacturer’s instructions (RaKaRa, Kyoto, Japan). The RNA concentration was measured spectrophotometrically at 220 nm using a spectrophotometer (Nanodrop 1000; Thermo Scientific, Waltham, MA, USA). RNA was digested with DNase I (RaKaRa, Kyoto, Japan), and reversely transcribed into cDNA with using PrimeScript^™^ RT reagent kit (RaKaRa, Kyoto, Japan). qRT-PCR was conducted in an Applied Biosystems 7500 Real-Time System (Bio-Rad, Hercules, CA, USA) using the SYBR^®^ Premix Ex TaqTM (Takara, Kyoto, Japan). The *nifH* primers were chosen for qRT-PCR (Roesch, Mergel & Bothe, 2002). The plant housekeeping gene *actin* was used as plant internal control (Padaria et al., 2014). The relative expression of the target genes was analyzed according to the standard comparative C(t) method (Livak & Schmittgen, 2001).

### Statistical analysis

One-way analysis of variance (ANOVA) was conducted using SPSS 20.0 (SPSS Inc., Chicago, IL, USA). The Pearson’s correlation coefficient was analyzed using GraphPad Prism 7.00 (GraphPad Software Inc., San Diego, CA, USA). Graphs were prepared using GraphPad Prism 7.00 and SigmaPlot software version 12.5 (Systat Software Inc., San Jose, CA, USA).

## Results

### Plant growth parameters

**Figure 1:**
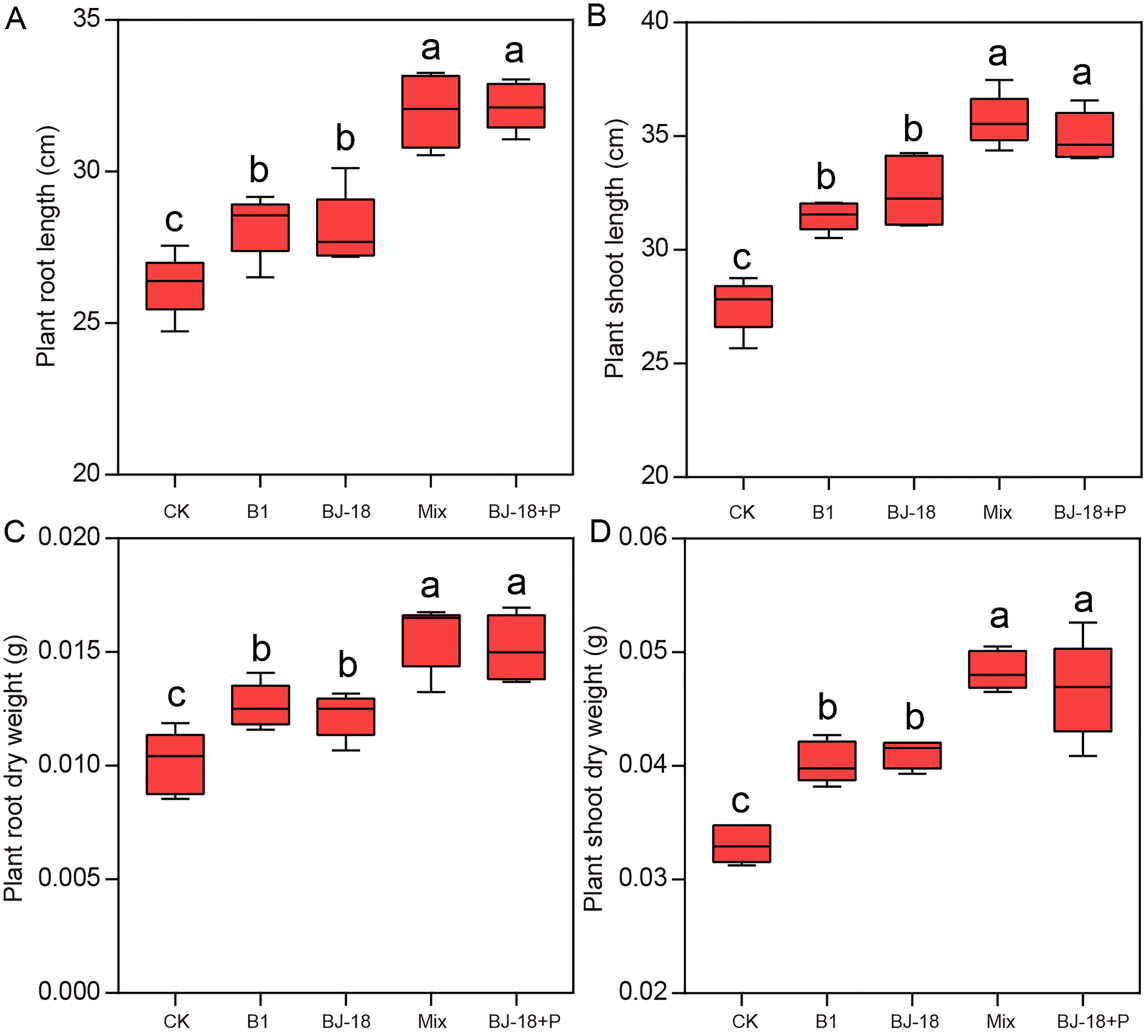
Wheat root length (A), shoot length (B), root dry weight (C) and shoot dry weight (D). Values are given as mean of five independent biological replicates, and bearing different letters (a, b, c) are significantly different from each other according to the least significant difference (LSD) test (*P* < 0.05). The bars represent the standard error. CK, control treatment; B1, Inoculation with *Paenibacillus* sp. B1; BJ-18, Inoculation with *P. beijingensis* BJ-18; Mix, Inoculation with *Paenibacillus* sp. B1 and *P. beijingensis* BJ-18; BJ-18+P, Inoculation with *P. beijingensis* BJ-18 and application KH_2_PO_4_.

Compared to CK treatment, B1, BJ-18, Mix and BJ-18+P treatments significantly increased wheat root length (Fig. 1A; 8%, 7%, 22% and 23%) and shoot length (Fig. 1B; 14%, 18%, 30% and 27%). However, Mix and BJ-18 treatments were not significantly different from each other in wheat length. Dry weight of wheat roots/shoots (Figs. 1C and 1D) in B1, BJ-18, Mix and BJ-18+P were significantly increased by 19%/25%, 16%/28%, 39%/49% and 42%/56%, respectively, compared to CK. For wheat dry weight, there was no significant difference between Mix and BJ-18+P treatments.

### Plant biochemical parameters

**Figure 2:**
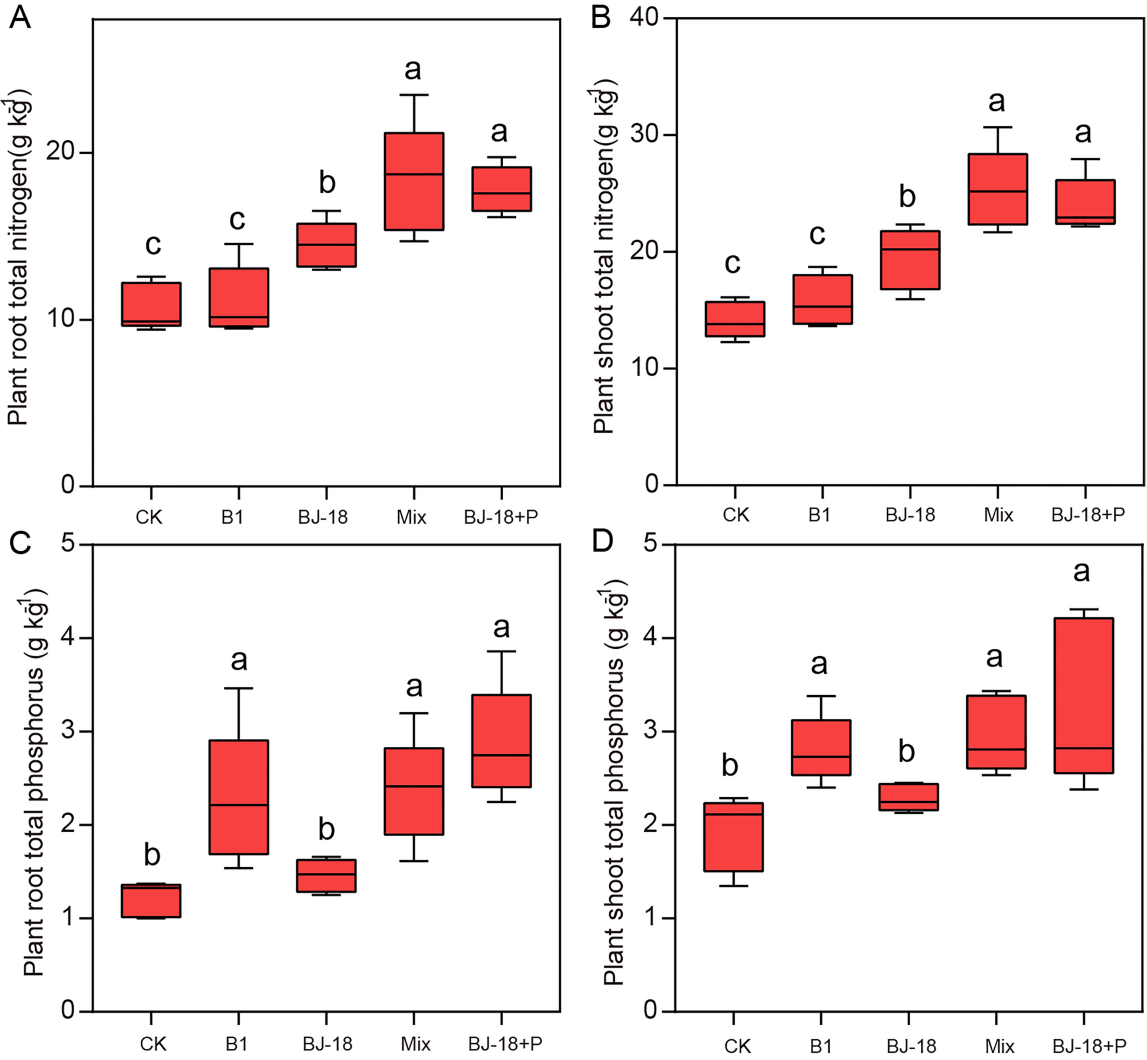
Plant root total N (A), shoot total N (B), root total P (C) and shoot total P (D). Values are given as mean of five independent biological replicates, and bearing different letters (a, b, c) are significantly different from each other according to the least significant difference (LSD) test (*P* < 0.05). The bars represent the standard error. CK, control treatment; B1, Inoculation with *Paenibacillus* sp. B1; BJ-18, Inoculation with *P. beijingensis* BJ-18, Mix: Inoculation with *Paenibacillus* sp. B1 and *P. beijingensis* BJ-18; BJ-18+P, Inoculation with *P. beijingensis* BJ-18 and application KH_2_PO_4_.

Total N of wheat roots in BJ-18, Mix and BJ-18+P were significantly increased by 35%, 71% and 36%, respectively, compared to CK treatment (Fig. 2A). Compared to CK treatment, total N of wheat shoots in BJ-18, Mix and BJ-18+P were significantly increased by 38%, 79% and 70%, respectively (Fig. 2B). Total P of wheat roots in B1, Mix and BJ-18+P were significantly increased by 88%, 95% and 136%, respectively, compared to CK treatment (Fig. 2C). Total P of wheat shoots in B1, Mix and BJ-18+P were significantly increased by 70%, 42% and 46%, respectively, compared to CK treatment (Fig. 2D). Compared to BJ-18 treatment, root total N, shoot total N, root total P and shoot total P in Mix treatment were significantly increased by 27%, 30%, 63% and 30%, respectively. There were no significant differences in plant N and P uptake between Mix and BJ-18+P treatments.

Compared to CK treatment, BJ-18, Mix and BJ-18+P treatments significantly increased SPAD value. Mix and BJ-18 treatments were not significantly different from each other in chlorophyll content (Table 1).

**Table 1:** Wheat chlorophyl content (SPAD value).

Treatments	SPAD value
CK	28.4 ± 0.4c
B1	28.2 ± 0.6c
BJ-18	29.9 ± 0.6b
Mix	32.4 ± 0.3a
BJ-18+P	32.1 ± 0.4a

**Note:**

Values are given as mean ± SE of five independent biological replicates and bearing different letters (a, b, c) are significantly different from each other according to the least significant difference (LSD) test (*P* < 0.05). CK, control treatment; B1, Inoculation with *Paenibacillus* sp. B1; BJ-18, Inoculation with *P. beijingensis* BJ-18; Mix, Inoculation with *Paenibacillus* sp. B1 *and P. beijingensis* BJ-18; BJ-18+P, Inoculation with *P. beijingensis* BJ-18 and application KH_2_PO_4_.

### Soil total N, available P and nitrogenase activity

**Figure 3:**
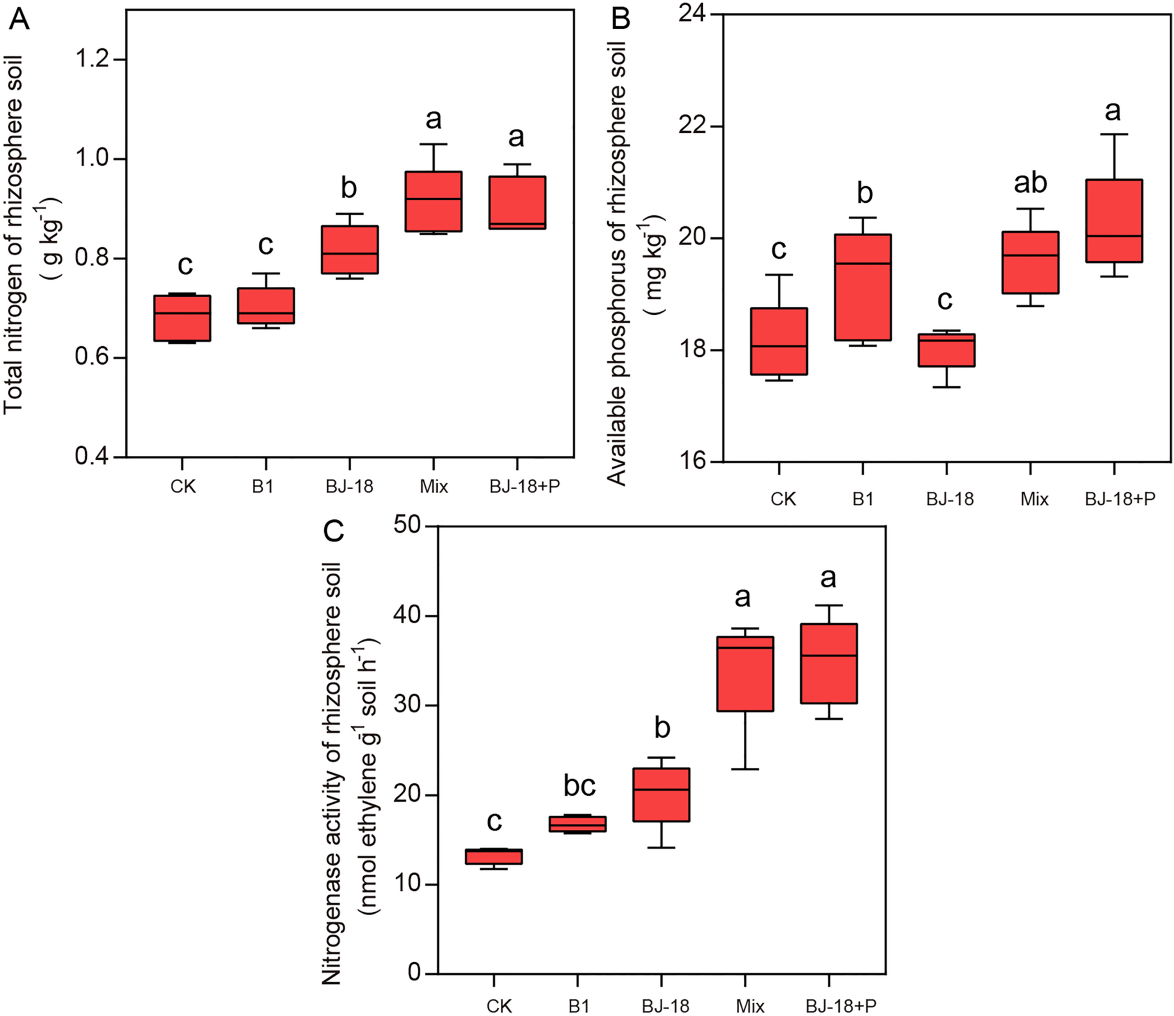
Rhizosphere soil total N (A), available P (B) and nitrogenase activity (C). Values are given as mean of five independent biological replicates, and bearing different letters (a, b, c) are significantly different from each other according to the least significant difference (LSD) test (*P* < 0.05). The bars represent the standard error. CK, control treatment; B1, Inoculation with *Paenibacillus* sp. B1; BJ-18, Inoculation with *P. beijingensis* BJ-18; Mix, Inoculation with *Paenibacillus* sp. B1 and *P. beijingensis* BJ-18; BJ-18+P, Inoculation with *P. beijingensis* BJ-18 and application KH_2_PO_4_.

Compared to CK treatment, treatments of BJ-18, Mix and BJ-18+P significantly increased total N of rhizosphere soil by 20%, 34% and 33%, respectively (Fig. 3A). B1, Mix and BJ-18 significantly increased available P in soils (Fig. 3B), by 6%, 8% and 12%, respectively, as compared to CK treatment. All treatments, B1, BJ-18, Mix and BJ-18+P, significantly increased rhizosphere soil nitrogenase activity by 26%, 52%, 158% and 163%, respectively, as compared to CK treatment (Fig. 3C). Soil nitrogenase activity of B1 did not differ significantly from the CK treatment. Mix treatment also showed much higher total N, available P and nitrogenase activity of rhizosphere soil than BJ-18 treatment. There were no significant differences in soil total N, available P and nitrogenase activity between Mix and BJ-18+P treatments.

### Transcript levels of *nifH* gene

**Figure 4:**
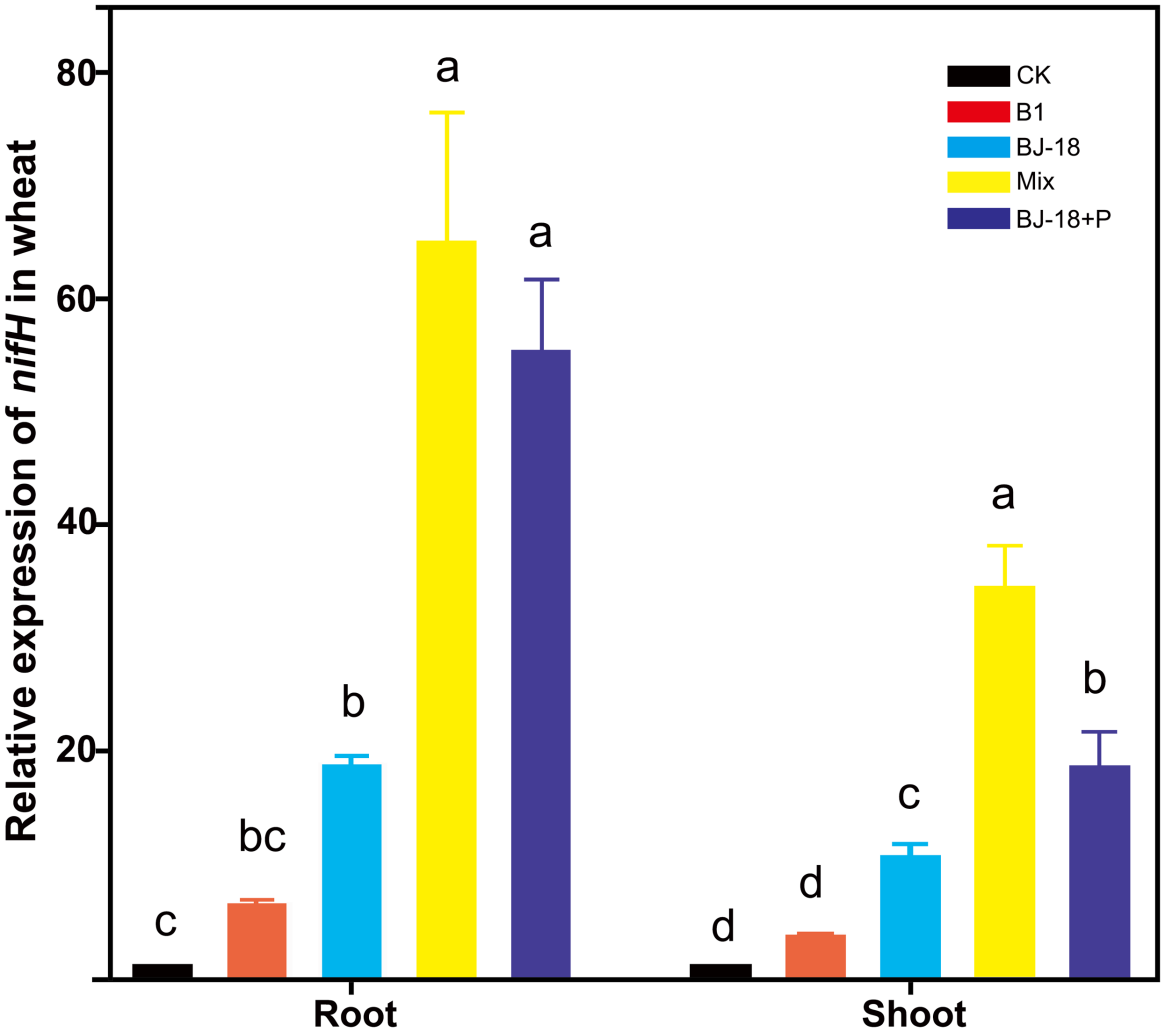
The relative expression of *nifH* in the host plant tissues. Values are given as mean of five independent biological replicates, and bearing different letters (a, b, c) are significantly different from each other according to the least significant difference (LSD) test (*P* < 0.05). The bars represent the standard error. CK, control treatment; B1, Inoculation with *Paenibacillus* sp. B1; BJ-18, Inoculation with *P. beijingensis* BJ-18; Mix, Inoculation with *Paenibacillus* sp. B1 and *P. beijingensis* BJ-18; BJ-18+P, Inoculation with *P. beijingensis* BJ-18 and application KH_2_PO_4_.

Transcripts of *nifH* were significantly up-regulated in wheat roots of BJ-18, Mix and BJ-18+P treatments by 18, 63 and 54 fold, respectively, and in wheat shoots of BJ-18, Mix and BJ-18+P treatments by 9, 31 and 17 fold, respectively, compared to those in the CK treatment (Fig. 4). Furthermore, treatment with Mix doubled transcription of *nifH* in wheat roots relative to treatment with BJ-18 alone.

### Relationships between biological nitrogen fixation and phosphorus content

**Figure 5:**
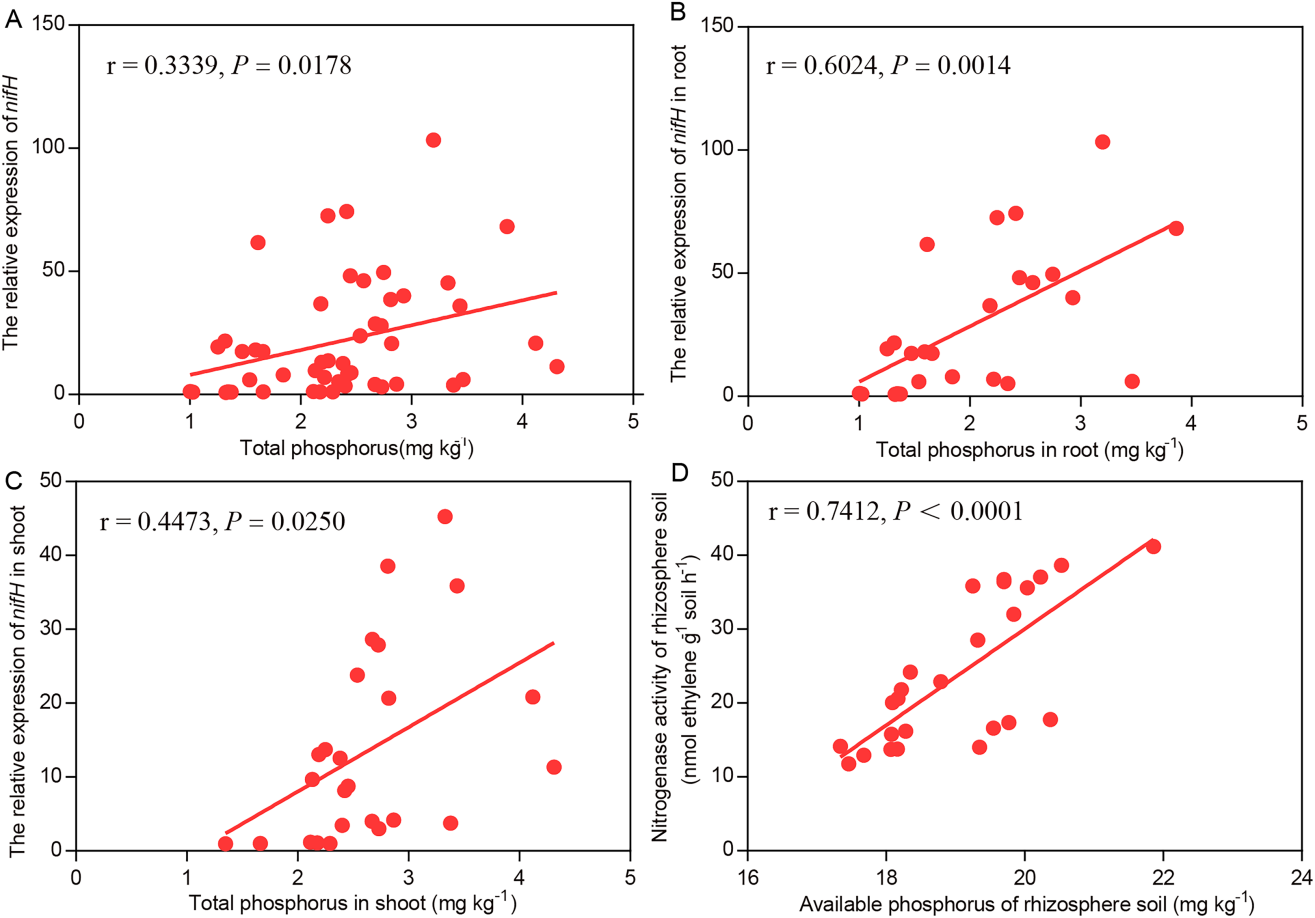
Pearson’s correlations between the efficiency of BNF and P nutrition of plant whole data set (A), plant root (B), plant shoot (C) and rhizosphere soil (D). *r* and *P* values are provided for each of the linear regression models.

In the Mix treatment, higher soil available P, soil nitrogenase activity, plant total P and *nifH* transcript levels were observed than in the BJ-18 treatment. For the data as a whole, the *nifH* transcript levels were strongly and positively correlated with P content in wheat biomass (Fig. 5A). For the data of wheat roots (Fig. 5B) and shoots (Fig. 5C), the *nifH* transcript levels were significantly positively correlated with plant total P content. In the rhizosphere soil, nitrogenase activity was strongly and positively correlated with soil available P content (Fig. 5D).

## Discussion

### Plant growth promotion

Co-inoculation with this particular P-solubilizing strain improved the efficacy of a diazotrophic PGPR, in terms of plant growth and nutrition. Inoculation with P solubilizing bacteria can significantly promote plant growth, and allows for reduced fertilizer application rates without sacrificing yield (Mehta et al., 2015; Jilani et al., 2007). Diazotrophic bacteria inoculation can increase plant biomass and nutrient status in potato, bamboo and maize through BNF (Naqqash et al., 2016; Xie et al., 2016; Ke et al., 2019). In both greenhouse and field conditions, co-inoculation of P solubilizing bacteria and diazotrophic bacteria can significantly promote wheat growth and nutrient uptake (Kumar et al., 2017; Sogut & Cig, 2019). Increased plant growth corresponded to increased SPAD values (Table 1). SPAD values positively correlated with leaf chlorophyll content, which is a crucial indicator of plant internal N nutritional status (Sibley et al., 1996; Chapman & Baretto, 1997).

### Are wheat rhizosphere BNF and soil available P related?

In this study, only one pair of P solubilizing bacterium and diazotrophic bacterium was studied, and since the soil used in greenhouse study was not sterile, soil nitrogenase activity observed in this study could have been in part due to indigenous diazotrophic bacteria in that soil. This increase of nitrogenase activity is comparable to the effects observed under other settings. P application doubled soil microbial N and soil N fixation rate in a clover-dominated sward and a restored prairie, respectively (Edwards, McCaffery & Evans, 2006; Reed et al., 2007). Yelenik, Perakis & Hibbs (2013) also reported that P limits BNF in the Douglas-fir series of the Klamath Region. In both greenhouse and field conditions, co-inoculation diazotrophic *Enterobacter* sp. and P solubilizing bacterium *Serratia marcescens* significantly promoted N and P uptake of wheat compared to single inoculation with diazotrophic bacteria or P solubilizing bacteria (Kumar et al., 2017).

### Are wheat endosphere BNF and plant P nutrition related?

The ability of PGPR strains B1 and BJ-18 to form close associations with plant roots, as established with observation of GFP-tagged constructs (Li et al., 2017, 2019a), could explain the synergy reported herein. Endophytes interact closely with plant hosts and readily exert beneficial traits (Santoyo et al., 2016). Endophytic diazotrophic *Klebsiella* was isolated from various plants (Govindarajan, Kwon & Weon, 2007; Liu et al., 2017). Endophytic diazotroph *Paenibacillus polymyxa* P2b-2R was isolated form pine stem, reduced seedling mortality, provided plant N by BNF, and increased plant dry weight (Anand, Grayston & Chanway, 2013). *Pseudomonas stutzeri* A15, isolated from rice rhizosphere and endosphere, promotes the growth and development of rice seedlings through BNF (Pham et al., 2017). Divito & Sadras (2014) reported that high plant P content was required for symbiotic BNF in legumes. In the legume–rhizobial symbiosis, arbuscular mycorrhiza stimulated BNF of symbiotic diazotrophic bacteria by improving plant P nutrition (Pueschel et al., 2017). In our study there was a significant positive correlation between asymbiotic *nifH* expression of endophytic diazotrophic bacteria and plant total P, both in the plant as a whole and in plant roots and shoots. Although there are no other reports on the effects of non-leguminous plant P nutrition on *nifH* expression by endophytes, our data showed that inoculation with the P solubilizing bacterium *Paenibacillus* sp. B1 provided positive BNF-feedback to endophytic diazotrophic bacteria by improving plant P nutrition. Therefore, we infer that *P. beijingensis* cells in plant tissues sensed the P signal and then regulated *nif* expression according to change of P levels.

## Conclusions

This study demonstrated that co-inoculation of a P solubilizing bacterium and a diazotroph can increase plant biomass and improve plant nutrition and improve rhizosphere soil physicochemical properties (total N, available P, nitrogenase activity) and the relative expression of *nifH* in plant tissues. This suggests that application of pairings of P-solubilizing and diazotrophic bacteria can improve the sustainability of agriculture.

## Supplemental Information

10.7717/peerj.9062/supp-1 Supplemental Information 1 Raw data.

## Additional Information and Declarations

### Data Availability

The following information was supplied regarding data availability: The raw measurements are available as a Supplemental File.
